# Physiological and Biochemical Response of Tropical Fruits to Hypoxia/Anoxia

**DOI:** 10.3389/fpls.2021.670803

**Published:** 2021-07-16

**Authors:** Noureddine Benkeblia

**Affiliations:** Department of Life Sciences, The Biotechnology Centre, The University of the West Indies, Kingston, Jamaica

**Keywords:** tropical fruits, physiology, biochemistry, hypoxia, anoxia

## Abstract

Aerobic respiration and oxygen consumption are indicators of routine metabolic rate, and dissolved oxygen in plant tissues is one of the most important environmental factors affecting their survival. The reduction of available O_2_ leads to hypoxia which causes a limitation of the oxidative phosphorylation; when O_2_ is absent, tissues generate ATP by activating the fermentative glycolysis to sustain glycolysis in the absence of mitochondrial respiration, which results in the production of lactate. Overall, hypoxia was reported to often decrease the respiration rate (O_2_ uptake) and delay the climacteric rise of ethylene in climacteric fruits by inhibiting action, thus delaying their ripening. Much research has been done on the application of postharvest hypoxia and anoxia treatment to temperate fresh crops (controlled or modified atmosphere), however, very few reported on tropical commodities. Indeed, the physiological mode of action of low or absence of oxygen in fresh crops is not well understood; and the physiological and biochemical bases of the effects low or absence of O_2_ are also yet to be clarified. Recent investigations using omics technologies, however, have provided useful information on the response of fresh fruits and vegetables to this abiotic stress. The aims of this review are to (i) report on the oxygen exchange in the crops tissue, (ii) discuss the metabolic responses to hypoxia and anoxia, and (iii) report the physiological and biochemical responses of crops tissues to these abiotic stresses and the potential benefits of these environmental conditions.

## Introduction

From the botanical point of view, tropical fruits are a diverse group of commodities native to tropical regions which are geographically defined as regions between the latitudes 23° North and South of the equator, with temperatures averaging around 27°C and little variation in photoperiod (Samson, 1986). Tropical fruits present a large biodiversity varying in structure, characteristics, and physiology (Wongs-Areea and Noichinda, 2014). Although the variations of tropical fruits are not well established, banana, pineapples, papaya, and avocado fall within the category of major tropical fruits, while others such as lychee, durian, rambutan, guava, passionfruit, mangosteen, tamarind, and some others are considered minor tropical fruits (FAO, 2003; Paull and Duarte, 2011, 2012).

Atmosphere composition consists of 78% nitrogen (N_2_), 21% oxygen (O_2_), 0.04% carbon dioxide (CO_2_), 0.93% argon, small amounts of other gases, and variable amount of water vapor at 20°C and absolute pressure of 1 atm. Oxygen depletion or hypoxia occurs when the partial pressure of oxygen is low enough to limit the production of ATP by mitochondria, whereas anoxia (absence of O_2_) is attained when ATP mitochondrial production is insignificant compared to that generated by glycolysis and fermentation. Under normal condition of oxygen level (*normoxia*) cells run aerobic respiration and energy in the form of ATP is produced by oxidative phosphorylation. However, a reduction in oxygen (*hypoxia*) reduces the oxidative phosphorylation, while the absence of oxygen (*anoxia*) stops the phosphorylation process (Wongs-Aree and Noichinda, 2018; Salvatierra et al., 2020). This diverts the production of energy to the fermentation pathway producing fermentative by-products that accumulate in the cells (Boersig et al., 1988; Pfister-Sieber and Brändle, 1994; Cho et al., 2021).

In plant cells, oxygen partial pressure between organs of plant, for example in shoots O_2_ concentration is much higher in comparison with roots (Kotula et al., 2015; van Dongen and Licausi, 2015). On the other hand, oxygen availability also varies significantly in time and space, and the distribution of active O_2_ depends on its diffusion and convection, and the conductivity of gas transport in specific tissues (Armstrong et al., 2006; Ho et al., 2011; Licausi, 2011; Wang et al., 2017).

Intrinsically, the oxygen status of cells is variable and depends to a great extent on the concentration or partial pressure of atmospheric oxygen supply. This status differentiates between hypoxia and anoxia. Tissues are under hypoxic conditions when the oxygen partial pressure is the limiting factor of ATP production; anoxic conditions are characterized by a limited production of ATP by oxidative phosphorylation, which is mainly produced by fermentation (Drew, 1997; Atwell et al., 2015).

**FIGURE 1:**
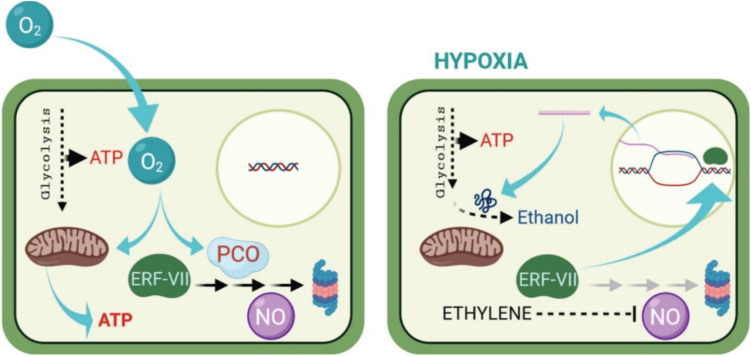
How plants sense oxygen. Under aerobic conditions (left), aerobic respiration in the mitochondria provides most off the energy (ATP) required for the cell metabolism. The ERF—VIII transcription factor genes are constitutively expressed, but their stability is compromised by the activity pf PCOs. Which is a process requiring oxygen, oxidize the N-terminal Cys residue, channeling the ERF-VII proteins to the proteasome, in a process also requiring nitric oxide (NO). Under hypoxia (right), the respiration in the mitochondria is drastically reduced, and AT production can only occur because of enhanced glycolytic activity. The ERF-VII proteins are stabilized because of the absence of oxygen and also thank to ethylene production, which dampers the presence of NO in the cell. The stable ERF-VII proteins migrate to the nucleus where they activate the transcription of Hypoxia-Responsive Genes (HRGs), including genes encoding proteins required for the alcoholic fermentation. (From Loreti and Perata, 2020, published under an open access Creative Common CC BY license).

Under unfavorable conditions of oxygen deprivation, plants develop different structural and metabolic adaptations that are genetically controlled, however, the form of adaptation and shift in metabolism depend on the specific response of each species and its tolerance (Figure 1; Beaudry, 2000; Huang et al., 2008; Ioannidi et al., 2009; Toro and Pinto, 2015; Boecx, 2018). This response causes the molecular mechanisms to react to the low or absence of oxygen (Davies et al., 1974; Jackson et al., 1991; Kennedy et al., 1992; Perata and Alpi, 1993; Ricard et al., 1994; Ratcliffe, 1995; Schmidt et al., 2018), as well as an acclimatization where ethylene was found to play a role (Figure 1; Hartman et al., 2021).

In plants, it has been established that anaerobic metabolic pathways other than ethanol production exist (Perata and Alpi, 1993; Ricard et al., 1994; Kolb and Joly, 2010; Ventura et al., 2020). First, plants respond to hypoxia/anoxia by producing lactate and the reaction consists of reducing pyruvate by the lactate dehydrogenase (LDH) (Mithran et al., 2010). Under prolonged exposure to hypoxia/anoxia, pyruvate is converted to acetaldehyde by pyruvate decarboxylase (PDC), and then the acetaldehyde is converted to ethanol by the acetaldehyde dehydrogenase (LDH). The lactate-ethanol transition pathway depends on the initial pH of the cytoplasmic compartment (Felle, 2005, 2010; Hossain and Uddin, 2011), and the lower the pH, the faster this transition occurs because LDH has an optimal alkaline pH of 8.0 while PDC has an optimal acidic pH of 5.8 (Davies et al., 1974; Davies, 1980; Morrell et al., 1990; Fox et al., 1994; Kato-Noguchi and Morokum, 2007; Cukrov et al., 2016).

Since tolerance of fresh crops to hypoxia/anoxia is of great economic importance in postharvest science and modified atmosphere packaging (MAP) technology, numerous investigations have been carried out to elucidate the mechanisms underlying the effect of oxygen deprivation on the physiological, biochemical, and organoleptic parameters of crops and their shelf-life. This review aims to describe the effects of low oxygen availability -hypoxia/anoxia- on the physiology, the biochemistry and the quality attributes of tropical fruits in order to determine the optimal condition of MAP application in postharvest handling and storage of these commodities.

## Anoxia/Hypoxia and Cell Metabolic Changes

**FIGURE 2:**
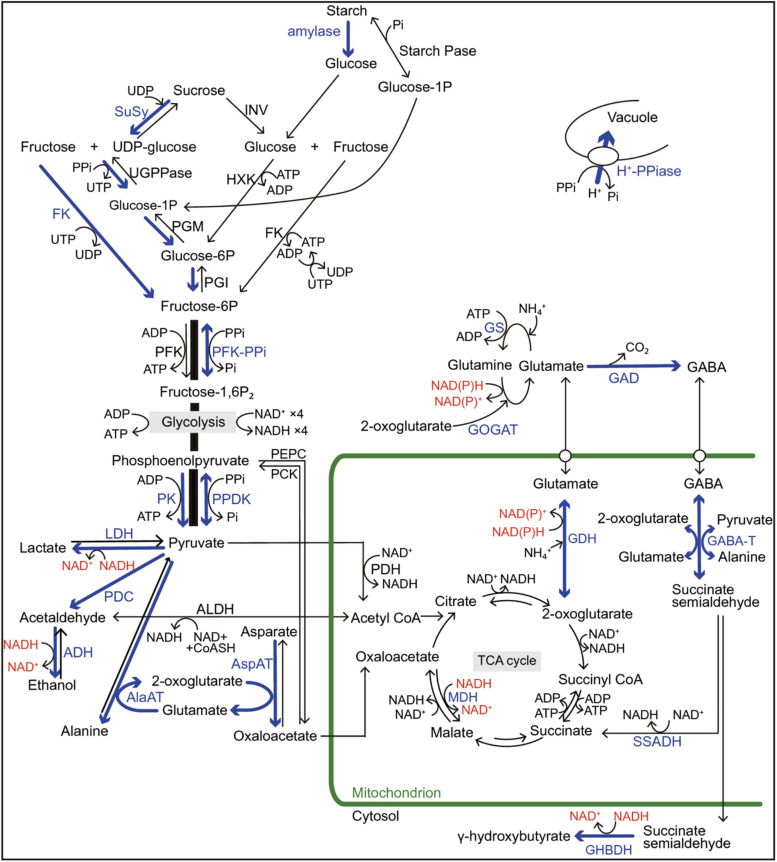
Regulations of sugar catabolism, fermentation, glycolysis, and major amino acid metabolism associated with NAD(P) + regeneration and ATP production in terrestrial and wetland plants under O_2_-deficient conditions. Blue arrows and letters indicate the reactions and enzymes in the up-regulated pathways when the mitochondrial electron transport and the TCA-cycle flux decrease under O_2_-deficient conditions. Red letters indicate the regeneration of NAD(P)+ from NAD(P)H. In rice plants, the blue pathways contribute to their tolerance to long-term O_2_ deficiency compared with the terrestrial plants. Some wetland plants such as rice also have a high ability to optimally regulate the pyruvate level by activation of pyrophosphate (PPi)-dependent phosphofructokinase (PFK-PPi) and pyruvate phosphate dikinase (PPDK) that consume PPi instead of ATP for energy conservation. Besides glycolysis, PPi is consumed to regulate the cytosolic pH by the tonoplast H+-pumping pyrophosphatase (H+-PPiase) instead of H+-ATPase in wetland plants. Although two independent pathways for sucrose degradation contribute to the regulation of glycolytic flux in both terrestrial and wetland plants, the UDP-dependent sucrose synthase (SuSy) pathway is regarded as energetically more advantageous for survival under O_2_-deficient conditions than the invertase (INV) pathway because here, PPi is utilized instead of ATP. Sugar supply to glycolysis through starch mobilization is observed in species with developed storage organs such as tuber, rhizome, and endosperm. In NAD(P)H regeneration during the metabolisms of 2-oxoglutarate and glutamate associated with γ-aminobutyric acid (GABA) production, the glutamate dehydrogenase (GDH) pathway without ATP consumption is more efficient in energy consumption than the NAD(P)H-dependent glutamine: 2-oxoglutarate aminotransferase (GOGAT) pathway with ATP consumption. The accumulation of some amino acids such as GABA, alanine, and glutamate play an important role in avoiding carbohydrate loss not only during O_2_-deficient conditions but also during the recovery phase of re-oxygenation after hypoxia/anoxia. Alanine accumulation by alanine aminotransferase (AlaAT) can operate non-circular TCA-cycle and gluconeogenesis under O_2_ deficiency and re-oxygenation. ADH, alcohol dehydrogenase; AlaAT, alanine aminotransferase; ALDH, acetaldehyde dehydrogenase; AspAT, aspartate aminotransferase; CoASH, coenzyme A; FK, fructokinase; GABA-T, GABA transaminase; GAD, glutamate decarboxylase; GHBDH, γ-aminobutyrate dehydrogenase; Glucose-1-P, glucose-1-phosphate; GS, glutamine synthetase; HXK, hexokinase; LDH lactate dehydrogenase; MDH, malate dehydrogenase; PCK, phosphoenolpyruvate carboxykinase; PDC, pyruvate decarboxylase; PDH; pyruvate dehydrogenase; PEPC, phosphoenolpyruvate carboxylase; PFK, ATP-dependent phosphofructokinase; PFK-PPi, PPi-dependent phosphofructokinase; PGI, phosphoglucoisomerase; PGM, phosphoglucomutase; Pi, phosphate; PK, pyruvate kinase; PPDK, pyruvate Pi dikinase; SSADH, succinate semialdehyde dehydrogenase; Starch Pase, starch phosphorylase; TCA, tricarboxylic acid; UDP, uridine diphosphate; UGPPase, UDP-glucose pyrophosphorylase; UTP, uridine triphosphate. (From Nakamura and Noguchi, 2020; an open access article distributed under the terms of the Creative Commons CC BY license).

Under hypoxic/anoxic conditions, the electron transport chain in the mitochondria leads to the progressive suppression and the inhibition of ATP synthesis. To compensate this lack of aerobic energy, the cell switches to produce ATP by anaerobic glycolysis. Basically, hypoxia/anoxia causes a decrease in ATP production, but this decrease is more significant in the anoxia-intolerant plants; this suggests that the ability of the anoxia-tolerant species to sustain their energy supply might be the key factor for survival under anoxia/hypoxia (Figure 2; Crawford, 1992; Hanhijärvi and Fagerstedt, 1994, 1995; Nakamura and Noguchi, 2020; Zahra et al., 2021). Nevertheless, response to oxygen deprivation is more complex than it seems and requires further investigation at different plants levels. Tolerance to hypoxia/anoxia, however, appears to depend on a dual morphological and metabolic adaptations which are specific to species and tissue types (Kennedy et al., 1992; Perata et al., 1993; Ratcliffe, 1995; Mariani and Ferrante, 2017; Nakamura and Noguchi, 2020; Zahra et al., 2021).

Although no fundamental metabolic differences have been observed between anoxia tolerant and intolerant plant species (Pfister-Sieber and Brändle, 1994), sugar availability is important since some tissues (e.g., roots) suffer more from sugar starvation under anoxia (Saglio, 1985). Indeed, when O_2_ becomes less available starch is rapidly hydrolyzed and channeled to the fermentative pathway for ATP production in order to compensate the lack of oxidative phosphorylation (Perata and Alpi, 1993). Although it was noted that starch reserve mobilization is affected by anoxia, anoxia-tolerant plants have been shown to break down starch much easier than anoxia-intolerant species (Perata et al., 1992).

From the metabolic point of view, oxygen unavailability and the production of ATP by fermentative glycolysis cause the acidification of the cytoplasm (Summers et al., 2000; Gout et al., 2001; Greenway and Gibbs, 2003) and, depending on the tolerance to low oxygen partial pressure, the cellular pH remains stable but drops when energy becomes short (Felle, 2005, 2010). This drop in the pH leads to the accumulation of lactate which in turn inhibits LDH and activates PDC producing acetaldehyde, which is converted to ethanol by alcohol dehydrogenase (ADH). However, cytoplasm acidification is not caused solely by lactate accumulation, but also by the possible passive H^+^ and potassium (K^+^) leakage from the vacuole and the protoplasm under limited ATP availability and inhibition of vacuolar H^+^-ATPase (Ratcliffe, 1995; Gout et al., 2001; Kulichikhin et al., 2007; Yemelyanov et al., 2020). Indeed, ethanol is the primary end product of fermentation in tissues of higher plants under to low oxygen, even though its catalysis and regulation involve components that will be identified in the future (Bui et al., 2019). However, other studies suggest that other fermentation pathways exist, such as the alanine pathway which is quantitatively minor (Davies et al., 1974; Nover, 1989; Jackson et al., 1991; Ricard et al., 1994). Under anaerobic fermentation, pyruvate can therefore be converted into products other than ethanol such as alanine, malate, and succinate which have been detected under early anoxic conditions. Although these diversified glycolytic pathways are recommended for enhancing the tolerance of plants to anoxia using molecular engineering (Lee et al., 2014; Diab and Limami, 2016), this physiological mechanism is not fully clear (Pfister-Sieber and Brändle, 1994).

Indeed, the effects of hypoxia/anoxia do not solely depend on the quantity of oxygen availability for ATP production, but also on the exposure time. Under prolonged anoxia, ethanol was found to be the most abundant end product of fermentation, but interestingly some plants showed their ability to release it into their surrounding environment, thereby increasing their tolerance to anoxia (Crawford and Zochowski, 1984; Kato-Noguchi and Morokum, 2007). Another metabolic consequence under anoxia is the alteration of the cellular redox state of the cell, and the ability of the plant to survive under hypoxia/anoxia consists of their capacity to maintain the cell redox (i.e., NADH/NAD^+^-ratio), since a decrease in NADH/NAD^+^ was noted in anoxia-intolerant plants (Chirkova et al., 1992; Nakamura and Noguchi, 2020). However, under oxygen deprivation anoxia-tolerant plants have higher ability to oxidize NADPH to NADP^+^ *via* glycolysis and fermentation, and this oxidation leads to less accumulation of reducing equivalents (Guglielminetti et al., 1995, 1999). Furthermore, prolonged hypoxia/anoxia, ATP needs also triggers the fermentation pathway and LDH and ADH generate NAD^+^.

From the molecular point of view, the mechanisms signaling the response to anoxia still remain unclear and not well elucidated, and the sensors of hypoxia/anoxia in plants are not clearly understood (Geigenberger, 2003; Gibbs and Greenway, 2003). Molecular responses to oxygen deprivation have focused on the regulation of genes expression and activation of enzymes involved in acclimation of metabolism such amylases (Bailey-Serres and Chang, 2005). So far, a direct oxygen sensor has not been established in plants, but some studies have demonstrated that increased ADH gene expression and fermentative metabolism are triggered under hypoxia/anoxia (Koch et al., 2000). Other mechanisms which use different sets of transcription factors (TFs) and ethylene responsive factor family (ERF) to perceive low-oxygen derived signals have been reported to play a primordial role in the determination of survival with reduced oxygen availability (Licausi et al., 2010b; Licausi, 2011).

Cytosolic calcium patterns (Ca^2+^)_*cyt*_ have also been recognized as important elements in signaling, and there has been increased interest in identifying the calcium fate involved in (Ca^2+^)_*cyt*_ changes in specific signaling pathways (Bush, 1995; Subbaiah et al., 1998; Yemelyanov et al., 2011; Lindberg et al., 2012; Hironari and Takashi, 2014). In this regard, one of the suggested hypotheses is the role of Ca^2+^ as a messenger. This hypothesis is supported by the work of Subbaiah et al. (1998) who observed an increase of Ca^2+^ in the cytosol suggesting its possible participation in anoxic signaling (Sedbrook et al., 1996; Bose et al., 2011). In a recent study, Igamberdiev and Hill (2018) suggested the possible release of Ca^2+^ from cell compartments to the cytosol under the decrease of ATP concentration resulting in the suppression of ATPases and activation of calcium ion channels.

## Effects of Hypoxia/Anoxia on the Respiration Rate and Ethylene Production

Unlike animal products, crops are living organisms and they continue to respire and are metabolically active during, either, before or after harvest. This respiration is the chemical process of energy production by converting glucose to carbon dioxide, water and heat. The respiration rate (*RR*) of a fresh crop is therefore determined by the speed at which this chemical process occurs, and a high *RR* leads to a faster depletion of glucose and loss of freshness. Indeed, under aerobic condition oxygen exchange in fruit depends greatly on its physiology (type of fruit and vegetable) (Platenius, 1942; Brady et al., 1970; Varoquaux et al., 1992; Kader, 1994) and it relies on O_2_ diffusion from the atmosphere to the fruit following Fick’s law (Palmes and Lindenboom, 1979; Kader and Saltveit, 2003).

However, as a dynamic process resulting from a chemical reaction, following van’t Hoff’s law (van’t Hoff, 1884; Kemp, 1987), *RR* is influenced by different factors mainly temperature (Waghmare et al., 2013). Atmospheric oxygen concentration also significantly affects *RR* depending on CO_2_ accumulation during respiration, regulation of the respiratory metabolism in addition to O_2_ diffusion limitation and availability which reduces significantly *RR* (Gupta et al., 2009; Ho et al., 2018). However, at low temperatures it was observed that *RR* was likely reduced by a specific response to a signal generated by a plant oxygen sensor (Ho et al., 2018).

From the metabolic point of view, more information is readily available on the effects of O_2_ than on CO_2_ (Watkins, 2000). Atmospheric carbon dioxide also influences the *RR* of crop commodities, however, the mechanisms on how CO_2_ affect *RR* are still not well understood, and CO_2_ might increase or decrease *RR* depending on the type of fruit (Mathooko, 1996). Overall, the *RR* of commodities decreases with the decrease in oxygen partial pressure below 21 kPa, and the effect of hypoxia becomes more significant below 10 kPa oxygen partial pressure (Chervin et al., 1996).

Practically, extensive literature has reported on the effects of hypoxia/anoxia on numerous temperate fruits (Ho et al., 2014; Boeckx et al., 2019), while limited references are readily available on tropical fruits in comparison. When exposed to low oxygen concentration, hypoxia/anoxia significantly reduced the *RR* of avocado (El-Mir et al., 2001), banana (Wade, 1974; Yi et al., 2006), Japanese persimmon (Imahori et al., 1998), mango (Yahia and Vazquez-Moreno, 1993), kiwi (Botondi et al., 2012; Huang et al., 2014), pear (Ho et al., 2018), and dragon fruit (Ho et al., 2021), while the absence of oxygen increased the *RR* of lychee (Liu et al., 2015).

On the other hand, oxygen is closely linked to the rate of respiration of fresh crops and is required for the biosynthesis of ethylene, and ERFs have been shown to play a role under hypoxia/anoxia (Licausi et al., 2010a,b; Licausi, 2011). In an interesting study, Sanders and de Wild (2003) modelized *in vivo* ethylene production rate in relation to O_2_ partial pressure using tomato, and their findings are in agreement with the molecular findings since they noted that ethylene biosynthesis and perception was positively related to O_2_ partial pressure (Mustroph et al., 2010). Thus, similar to the effects on *RR*, hypoxia/anoxia reduced levels of oxygen uptake and slowed down ethylene production in avocado (Zhang et al., 2011), saskatoon fruit (Rogiers and Knowles, 1998), banana (Imahori et al., 2013), and kiwi fruit (Botondi et al., 2012).

**FIGURE 3:**
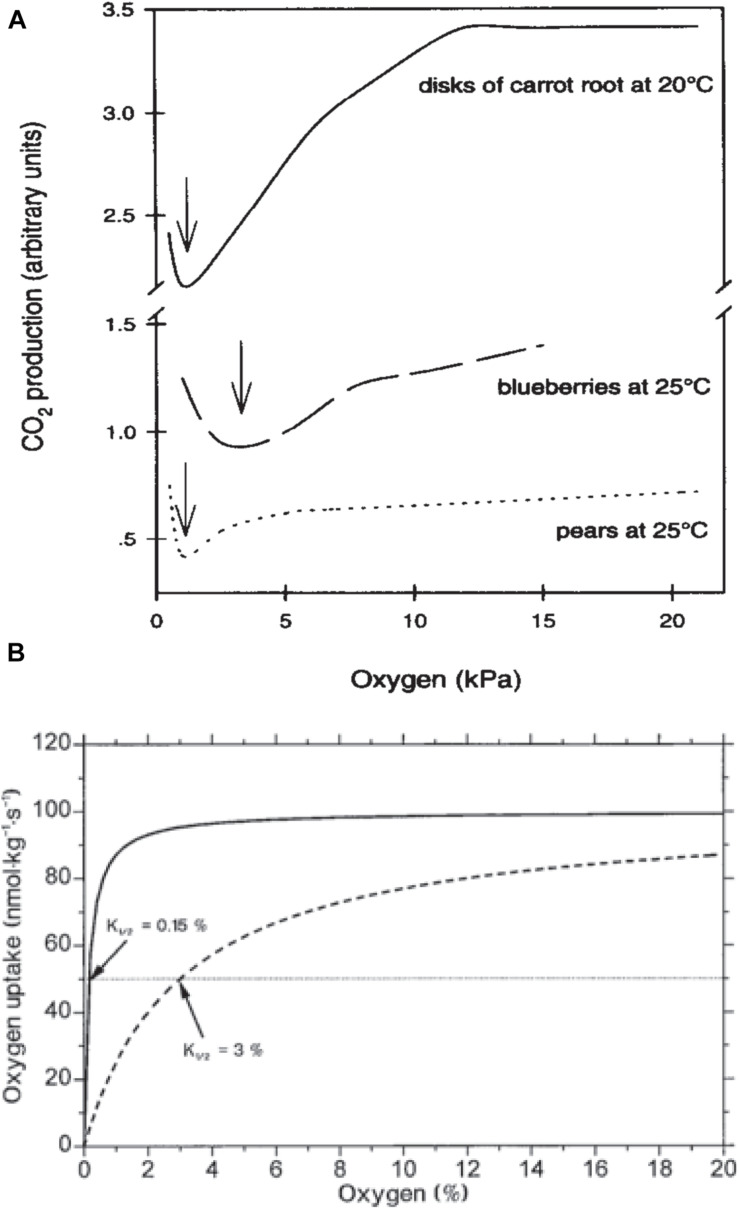
**(A)** Plant CO_2_ production in response to oxygen depletion. Data on carrot are from Leshuk and Saltveit (1990), on blueberries from Beaudry et al. (1992) and on pears from Boersig et al. (1988). Arrows indicate ACPs (Chervin et al., 1996, with permission of Elsevier). **(B)** Hypothetical respiratory responses to O_2_ for a respiratory system of low diffusive resistance exhibiting a K_1__/__2_ of about 0.15% O_2_, representative of single cells or tissues, and a tissue with significant diffusive resistance to gas exchange with an K_1__/__2_ of 3% O_2_ (with open permission).

Nevertheless, a decrease in the *RR* does not change the respiratory quotient (RQ = CO_2_/O_2_) –which is a good indicator of the trigger of the fermentation pathway– until O_2_ partial pressure reaches the compensation point c.a. 2 kPa (Figures 3A,B; Beaudry et al., 1992; Banks et al., 1993; Peppenlenbos et al., 1996). Therefore, this suggests that commodities are not significantly stressed by oxygen depletion since carbohydrates are still completely oxidized to CO_2_ and the end products of anoxia, namely lactate and acetaldehyde, do not accumulate until the oxygen partial pressure falls below 2 kPa (Beaudry et al., 1992).

## Hypoxia/Anoxia and Fresh Crops Ripening, Quality, and Storability

As described above, hypoxia/anoxia may have beneficial effects, such as slowing down the *RR*, ethylene production, and even reducing the incidence of some physiological disorders (Mojević and Tešanović, 2011; Lurie and Tonutti, 2014; Kongpatjirak et al., 2016) such as delaying ripening (Pesis et al., 1994; Pegoraro et al., 2012), softening (Wu et al., 2020), deastringency (Zhu et al., 2018), browning (Phonyiam et al., 2016), internal breakdown (Lizada and Rumbaoa, 2017), and also diseases (Fallik et al., 2003). However, it is important to note that following exposure of the fruit to anoxic conditions, the accumulation of the ethanol fermentation metabolites -acetaldehyde and ethanol- may be enhanced to extreme levels. The occurrence of anaerobic conditions in the internal atmospheres of fruit and vegetables, and their tendency to develop off-flavors also depend on their anatomical structure and morphology (Porat and Falik, 2008).

The modification of atmosphere was first developed by Kidd and West (1927a, b), and a general consensus was reached on hypoxia/anoxia and the effects in reducing rate of respiration, ethylene synthesis and sensitivity, and lipid catabolism and other oxidative reactions (Burton, 1974; Knee, 1991). Hypoxic/anoxic conditions have shown to delay ripening and senescence of many fruits and these effects were behind the development of MAP and controlled atmosphere (CA) postharvest storage technologies. Extensive literature is readily available on the effects of hypoxia/anoxia on crops, but little on tropical fruits. Green banana fruit stored under low (2%) and absence (0%) of oxygen for 7 days showed high ADH activity and accumulated more ethanol under total anoxia (0% O_2_), while low oxygen showed a delayed onset of the climacteric peak and extended the shelf-life of banana but reduced remarkably the production of ester volatiles, i.e., ethyl acetate, isoamyl acetate, and isobutyl acetate (Imahori et al., 2013). Similar results on banana were obtained and the application of pure nitrous oxide (100% N_2_O) or combined to low O_2_ levels delayed ripening and extended the shelf-life of the fruit (Palomer et al., 2005; Yi et al., 2006).

Short anoxic treatment of pineapple maintained flesh and pulp color; delayed the increase in total sugar content and enhanced total ascorbic acid content during storage; and maintained overall postharvest quality of the fruit stored at ambient temperature (Techavuthiporn et al., 2017). When hypoxically pre-treated (3% O_2_ for 24 h) or exposed to low oxygen (1 and 0.25% O_2_ for 1–3 days), avocado fruit increased their tolerance to hypoxia (El-Mir et al., 2001). Litchi is known to have a very short shelf-life, and its short exposure to anoxia (0% O_2_) markedly delayed skin browning and reduced rotting while maintaining the physical quality of the fruit during storage (Jiang et al., 2004; Liu et al., 2007). Mango is also one of the most widely consumed but perishable tropical fruit. Interestingly, anoxic treatment for 24 h prior to storage showed to be effective in retarding ripening, maintaining firmness, and delaying color change (Kongpatjirak et al., 2016). In a study on cherimoya, Palma et al. (1993) noted that hypoxia (5% O_2_) delayed ripening and the edible condition differed with oxygen treatment and was inversely proportional to O_2_ concentration. In a recent study, Ho et al. (2021) investigated the effect of different hypoxic treatments on dragon fruit (*Hylocereus undatus*) and their results showed that 2 kPa O_2_ + 5 kPa CO_2_ was the optimal hypoxic treatment in maintaining the shelf-life of the fruit during storage.

Another benefit of hypoxia/anoxia is the improvement of the quality attributes of some fruits. Hypoxia was shown to be a beneficial treatment for the removal of astringency from persimmon to improve the fruit quality after harvest. Although the longer the fruit is stored, the less effective is the treatment (Salvador et al., 2008), the application of hypoxic treatment with high CO_2_ (95%) effectively reduced high soluble tannins (SCTs) content which is one of the most important causes of persimmon fruit astringency (Min et al., 2014). Table 1 summarizes the main findings of hypoxia/anoxia treatments effects on tropical fruits.

**TABLE 1:** Effects of hypoxia/anoxia on some tropical fruits.

Fruit	Hypoxia/anoxia	Benefits	References
Avocado	2–5% O_2_	Reduce respiration rate Ethylene production Delay ripening	El-Mir et al., 2001
Banana	2–5% O_2_	Delay ripening	Palomer et al., 2005; Yi et al., 2006; Imahori et al., 2013
Cherimoya	5%	Reduce respiration rate Reduce ethylene production Delay ripening Delay ripening Firmness retention	Palma et al., 1993; Escribano et al., 1997
Durian	3–5% O_2_	Reduce CO_2_ production Reduce ethylene production Delay ripening	Minh, 2017
Litchi	5%	Reduce skin browning	Jiang et al., 2004; Liu et al., 2007
Mango	3–5% O_2_	Delay ripening	Kongpatjirak et al., 2016
Papaya	2–8% O_2_	Delay ripening Degreening and softening Enhanced quality	Rohani et al., 1997; González-Aguilar et al., 2003
Pineapple	2–5% O_2_	Reduce respiration rate Delay senescence	Techavuthiporn et al., 2017
Rambutan	3% O_2_	Reduce respiration rate Delay senescence	O’Hare et al., 1994
Sweetsop	3–5 O_2_	Reduce respiration rate Reduce ethylene production Delay ripening	Broughton and Guat, 1979; Venkatram et al., 2016
Dragon	2% O_2_	Delay senescence	Ho et al., 2021
Persimmon	95% CO_2_	Removal of astringency	Min et al., 2014; Salvador et al., 2008

## Conclusion and Future Directions

In postharvest science, hypoxia/anoxia treatments combined with packaging technology have been used as one of the most inexpensive non-chemical technology to prevent physiological disorders, reduce diseases incidence, delay ripening, extending shelf-life, and even improving some quality attributes of a large number of temperate fresh commodities during transportation and storage. Nevertheless, few studies have been focusing on how oxygen deprivation affects tropical fruits. These atmospheric conditions and treatments with low or total absence of oxygen have shown different efficacy levels which depend on composition of the atmosphere, the treatment and storage duration, and the type of commodity treated and their tolerance and response to total or partial absence of oxygen. Overall, on several tropical fruits low or absence of oxygen has shown its involvement in reducing the *RR* and ethylene biosynthesis, prolonging therefore the shelf-life of these commodities and maintaining some quality attributes and freshness. However, data showed that changes in oxygen availability and ethylene emission rates in reaction to the surrounding hypoxic or anoxic atmosphere varies with different intrinsic and extrinsic factors particularly oxygen levels and temperatures. On the other hand and from the fundamental point of view, the timing of the converging pathways to acetaldehyde and ethanol production and the distinct energy-dependent signaling pathways operating during hypoxia/anoxia are still unclear and not fully understood since most tropical fruits are known to have higher *RR*s and many of them are climacteric fruits. Besides the progress and advances in plant physiology and hypoxia/anoxia effects on plant tissues, fruits admittedly react differently to extremely low or total absence of oxygen conditions during exposure and storage in terms of tolerance, *RR*, ethylene production, volatiles biosynthesis, and acetaldehyde, alanine, and ethanol production and accumulation. In order to decipher the effects of hypoxia/anoxia mechanisms on tissues in general, it is first crucial to elucidate the different oxygen sensing mechanisms involved, the specific molecular and metabolic changes occurring at the earliest stages of the hypoxic/anoxic conditions, and establish whether these changes are a sort of adaptation response to oxygen deprivation. Another direction in understanding hypoxia/anoxia effects on plants is to elucidate whether the different oxygen-sensing mechanisms have different activation thresholds, and how these sophisticated sensing and signaling networks likely enable plants to tailor their adaptive responses to face the severity and duration of hypoxic/anoxic conditions. Elucidating these mechanisms will be of great importance and applications in enhancing our understanding of crop physiology in these extreme conditions. It will also a key step in determining which low oxygen atmospheric conditions may be worth testing on tropical fruits to determine the optimal postharvest handling and storage conditions of these sensitive commodities and maintaining longer their freshness and quality attributes. Indeed, anoxia/hypoxia should be further investigated for its potential application to extend the shelf-life and preserve the quality attributes of numerous less known tropical fruits by setting optimal O_2_ concentration either as a pre-treatment for a very short time span prior to storage or in MAP or CA storage technologies.

## Author Contributions

The author confirms being the sole contributor of this work and has approved it for publication.

## Conflict of Interest

The author declares that the research was conducted in the absence of any commercial or financial relationships that could be construed as a potential conflict of interest.
